# Advances in Hyaluronic Acid for Biomedical Applications

**DOI:** 10.3389/fbioe.2022.910290

**Published:** 2022-07-04

**Authors:** Aqeela Yasin, Ying Ren, Jingan Li, Yulong Sheng, Chang Cao, Kun Zhang

**Affiliations:** ^1^ School of Materials Science and Engineering, and Henan Key Laboratory of Advanced Magnesium Alloy and Key Laboratory of Materials Processing and Mold Technology (Ministry of Education), Zhengzhou University, Zhengzhou, China; ^2^ School of Materials Science and Engineering Henan University of Technology, Zhengzhou, China; ^3^ Department of Cardiology, The First Affiliated Hospital of Zhengzhou University, Zhengzhou, China; ^4^ School of Life Science, Zhengzhou University, Zhengzhou, China

**Keywords:** hyaluronic acid, properties and biomedical application, injectable hydrogels, target nanoparticles, coatings

## Abstract

Hyaluronic acid (HA) is a large non-sulfated glycosaminoglycan that is the main component of the extracellular matrix (ECM). Because of its strong and diversified functions applied in broad fields, HA has been widely studied and reported previously. The molecular properties of HA and its derivatives, including a wide range of molecular weights but distinct effects on cells, moisture retention and anti-aging, and CD44 targeting, promised its role as a popular participant in tissue engineering, wound healing, cancer treatment, ophthalmology, and cosmetics. In recent years, HA and its derivatives have played an increasingly important role in the aforementioned biomedical fields in the formulation of coatings, nanoparticles, and hydrogels. This article highlights recent efforts in converting HA to smart formulation, such as multifunctional coatings, targeted nanoparticles, or injectable hydrogels, which are used in advanced biomedical application.

## 1 Introduction

In 1934, hyaluronic acid (HA) was first discovered in vitreous eyes of cows by Karl Meyer and John Palmer (Prestwich, 2011). The subsequent study found that it was naturally present in all vertebrate animals and human beings (Filion and Phillips, 2002) and almost originated in all body tissues and fluids, such as hyaline cartilage, synovial fluid, and the eye vitreous humor (Huynh and Priefer, 2020). In the 1950s, the chemical formula of HA was solved, and it was first isolated as an acid but under physiological conditions (Nosenko et al., 2020). The HA structure is an un-branched and non-sulfated glycosaminoglycan that comprises N-acetyl D-glucosamine and D-glucuronic acid repeatedly linked *via* glycoside bonds in the arrangement of alternating β-(1 → 4) and β-(1→ 3) bonds (Bukhari et al., 2018; Dovedytis et al., 2020). HA is a biodegradable polymer component of the extracellular matrix (ECM) which has a wide range of molecular weights about 10^3^ to 10^7^ Dalton with an outstanding ability to trap 1,000 times the weight of water (Salwowska et al., 2016), leading to its good lubrication with body and high viscosity. It plays a major role in water transport and tissue hydration because of its extremely high water-holding capability (Li et al., 2021a). Because of the high water-holding capability, HA is known to fix the negative charge of high density in its chain from the carboxyl groups, which causes the water molecules to be retained in its structure and maintains osmotic pressure (Huang and Huang, 2018a).

In 1942, HA was first used at a commercial level and then widely applied in bioorganic, biochemistry, radiochemistry, molecular biophysics, and compound polymer chemistry (Lee et al., 2012). In recent years, HA and its derivatives have been made into various formulations, such as coatings, hydrogels, and nanoparticles because of the benefit of its properties such as anti-coagulant, anti-inflammatory, antiproliferative, immunomodulatory, targeted transport, sustained-release, and cell compatibility and have been systematically applied in tissue engineering, wound healing, anticancer treatment, and cosmetics (Burdick and Prestwich, 2011; Khunmanee et al., 2017; Séon-Lutz et al., 2019). A very interesting example is that HA is an irreplaceable ingredient in all cosmetics at present. It is used in all cosmetic products having skin-protective effects due to its properties of anti-aging. Moreover, HA results in the skin being softer, radiant, and smoother, which are attributed to its capability of replenishing moisture and holding water (Altman et al., 2019). HA is used for all types of skin because it is non-toxic, non-allergic, and non-sensitizing (Sahiner et al., 2020). In addition, it also participates in several important biological functions, such as manipulation, motility, differentiation and proliferation of skin cells, and biomechanical properties (Omer et al., 2019).

The molecular structure and characteristics of HA and its applications have been widely reported. This article focused on the newly discovered functions of HA and its derivatives, which resulted in formulations such as coatings, nanoparticles, and hydrogels and the novel application in tissue engineering, wound healing, cancer treatment, ophthalmology, and cosmetics of these formulations.

## 2 Biomedical and Molecular Properties of Hyaluronic Acid

HA plays numerous structural tasks throughout the ECM by specific and non-specific interactions, which makes it a vital factor for cellular transformation and proteins with receptors and particular molecules (Arulmoli et al., 2016). A few examples of the molecules and receptors are the lymphatic vessel endothelial HA receptor (LYVE), neurocan, receptor for hyaluronan-mediated motility (RHAMM), glial HA-binding protein (GHAP), and cluster determinant (CD44) (Kim et al., 2018). HA plays a main role in the development of macrophages, eosinophils, epithelial cell tissues, and many other cells. It is also important in the formation of scars and healing: in the presence of lower molecular weight HA, less scarring tissue is determined, while a high relative molecular mass is determined in healing wounds. These results highlight the relationship between the relative molecular mass of HA and the scarring and healing process (Graça et al., 2020). The finding also suggests that HA with a high molecular weight favored tissue integrity and cell quiescence, whereas developing HA fragments (low molecular HA, 10^3^ Da) signaled injury and initiated the inflammatory response (Sato et al., 2016).

HA fragments are involved in the stimulation of angiogenesis and support the proliferation of fibroblasts. Pro-antigenic properties are exhibited particularly by short HA oligosaccharides comprising 6–20 saccharide units. HA oligosaccharide is mitogenic for endothelial cells (ECs), supports their migration, and includes multiple signaling pathways (Guarise et al., 2019). Their low toxicity and good biocompatibility make them an ideal therapeutic agent for the growth of new blood vessels. HA oligosaccharides are also ideal therapeutic agents for combating hair loss and stimulating hair growth. In the field of reproductive medicine, HA oligosaccharides can help screen high-quality sperms and improve the probability of pregnancy (Bermejo-Velasco et al., 2019): only mature sperms can combine with HA oligosaccharides to start the subsequent fertilization process. The binding of sperms to HA oligosaccharides is mainly influenced by the following three factors: the sperm chromosomes are complete; the DNA fragment rate of the sperms is low enough; and the shape of the sperm head is normal.

HA fragments can also be used for treating and preventing wrinkles, expression lines, fibroblastic depletion, and scars. HA oligosaccharides (with molecular weight <5 × 10^3^ Da) contribute to the suppression of wrinkles in both layers of the skin (Smejkalova et al., 2015). At the epidermis, they stimulate the endogenous high molecular HA, which has a positive effect on hydration. At the level of the dermis, HA decreases the creation of pro-inflammatory interleukins, which are responsible for the generation of free radicals capable of damaging both components of the skin cell and ECM themselves. HA is a natural moisturizing factor substance (Guarise et al., 2019). The more important advantage of HA compared with other moisturizers (polyethylene glycol, glycerol, ethylene glycol, propylene, and sorbitol) is that it is not affected by relative humidity (Smejkalova et al., 2015). Dissimilar to the other moisturizers, HA has good water retention capability both at high and low relative humidity.

## 3 Recent Hyaluronic Acid Applications in the Biomedical Field

**FIGURE 1:**
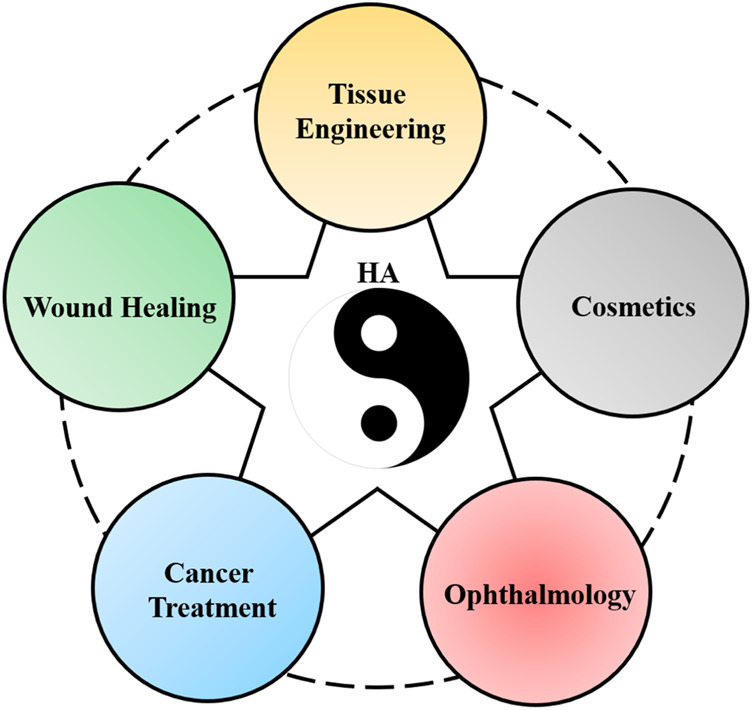
Traditional Chinese-five-element diagram is used to compare the application of HA in the following five biomedical aspects: the “Taiji” in the middle indicates HA; the “gold element” indicates the tissue engineering application of HA; the “wood element” indicates the wound healing application of HA; the “water element” indicates cancer treatment application of HA; the “fire element” indicates the ophthalmology application of HA; and the “earth element” indicates the cosmetics application of HA.

HA plays an important role in the medical field, and its advanced application is shown in Figure 1 by the analogy of “gold,” “wood,” “water,” “fire,” and “earth” in the traditional Chinese five elements. Among them, “Taiji” in the middle often symbolizes the coordinator of balance in ancient Chinese mythology, which refers to HA here. The “gold element” often stands for the blade, which refers to tissue engineering applications; the “wood element” often stands for repair and growth, which refers to wound healing applications here; the “water element” usually represents tenderness, but can overcome rigidity, which refers to cancer treatment applications; the “fire element” indicates “piercing eye,” which refers to ophthalmology applications; and the “earth element” indicates to moisten all things, which refers to cosmetics application.

### 3.1 Tissue Engineering

In 1988, the term tissue engineering was developed (Caló and Khutoryanskiy, 2015). It was defined as the replacement and improvement of specific organs or tissue engineering materials and synthetic strategies. Every year, there are millions of patients suffering from an organ failure or loss caused by diseases or accidents. In the U.S., more than eight million surgeries are performed to treat these patients (Chanda et al., 2018). The whole cost of this issue is estimated in the U.S. economy to be around about 400$ billion per year. Organ and tissue transplantations are usually conventional therapies, but they are significantly limited by contributor shortages (Bernhard and Vunjak-Novakovic, 2016). Hydrogels are an advanced application in tissue engineering as the scaffolds for cell loading, growth, differentiation, and delivery. HA hydrogels can provide a different formulation for tissue engineering which includes space-filling agents and mucoadhesive materials (Menaa et al., 2011). HA hydrogels have the ability to change the three-dimensional structures of the natural ECM, which is conducive to releasing more cytokines for the versatile design of tissue engineering (Chircov et al., 2018). The most importantly used group of HA hydrogels are space-filling agents for bulking as a biological glue to enhance the therapeutic effectiveness of the matrix or scaffold by preventing cell adhesion, which also improves anti-aging purposes. Another application of HA hydrogels is transplanting cells in the body for tissue repair and regeneration, including bone, cartilage, and smooth muscle (Nosenko et al., 2020). Regenerative medicine uses the technique of integrated strategies from tissue engineering to replace the tissues suffering from injury or diseases (Zhai et al., 2020), and HA hydrogels as the carriers of cells participate in tissue regeneration, and their biocompatibility (cell compatibility, blood compatibility, and histocompatibility) is an essential property. In addition, HA can act as delivery vehicles in an *in vitro* environment for the development of factors and is also able to elicit this response in an *in vivo* model (Fakhari and Berkland, 2013). It is generally found in various vitreous studies that different approaches to tissue engineering have important benefits. The regenerative tissues made of HA in different animal models may load autologous growth factors or allogeneic factors (Caló and Khutoryanskiy, 2015). To obtain better delivery ability for the factors, HA may be prepared with other biopolymers with different techniques.

### 3.2 Wound Healing

**FIGURE 2:**
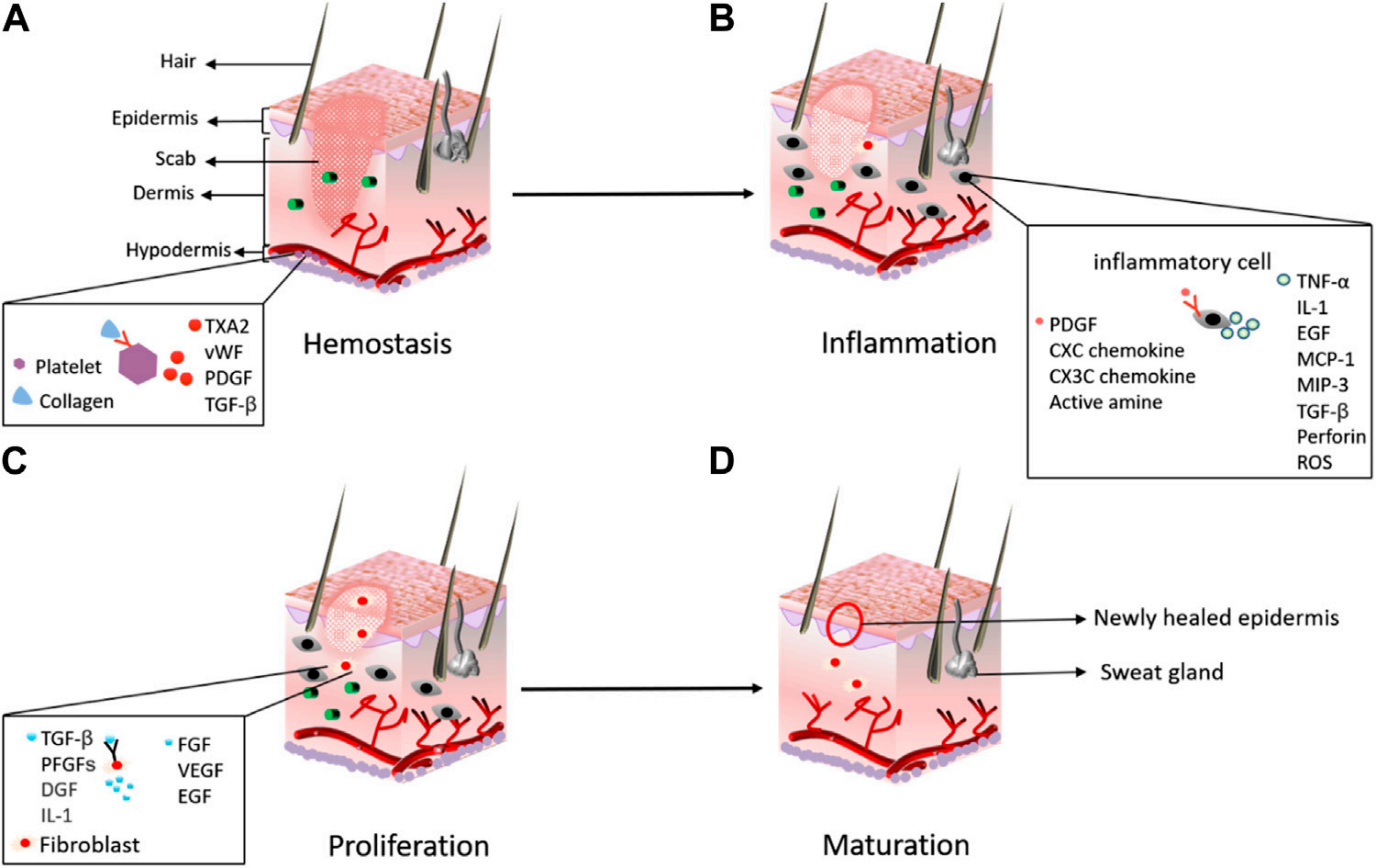
Major stages in the process of wound healing: **(A)** hemostasis; **(B)** inflammation; **(C)** proliferation; and **(D)** maturation (Su et al., 2021).

HA plays an important role in the different biological processes that are essential for wound healing. Wound healing is a chain of biological processes including granular tissue formation, inflammation, and the construction of the injured epithelium and its re-modeling (Figure 2) (Su et al., 2021). In the complete process, HA acts as a mediator, which is why it is used for wound healing in external skin injuries, scars, and pressure sores (Vasvani et al., 2020). HA is used in wound healing processes such as remodeling and formation of tissue. It also plays a multifaceted role in cellular and matrix events; thus, it is used for the treatment of chronic and acute wounds such as postoperative incision, abrasion, and burns (Brown and Jones, 2005). HA has many characteristics that other products lack that can be used for wound healing: it has no allergenic actions; however, it provides bio-stimulating and inflammatory effects in the regeneration process (Vasvani et al., 2020). The high compatibility of HA makes it possible to combine it with other drugs for all aspects of wound healing, such as surgery, nutritional ulcer, and burn healing and is especially useful in the period of the first 3 days of wound healing (Jin et al., 2010). The oxidized HA film base has the ability to accelerate the healing of stitches. During laparoscopy, HA is used for bowel perforation and to secure the defective serous membrane (Lim et al., 2012). Stomach and duodenum ulcers can also be treated using HA. Anti-ulceration of HA is associated with its capability to restrain H2 histamine receptors and the action of trypsin.

Because of the low molecular weight of HA, it is often used as a transporter of bioactive compounds. As explained earlier, HA has a high rate of turnover in biopolymers (Sato et al., 2016). Whenever tissue is injured, the existing quantity of HA undergoes the process of decomposition, which results in the appearance of an oligosaccharide wreckage that is implicated in the development of a transitory ECM (Bowman et al., 2018). Synthesis and accretion of HA with diverse molecular weights is speedily induced in the damaged area by activating genes and hyaluronidase. All uses of HA in wound healing that are mentioned previously explain that the series of macromolecules is a vigorous participant in the repairing process of damaged tissues, while HA is the smart carrier and coordinator of these macromolecular activities (Huynh and Priefer, 2020).

**FIGURE 3:**
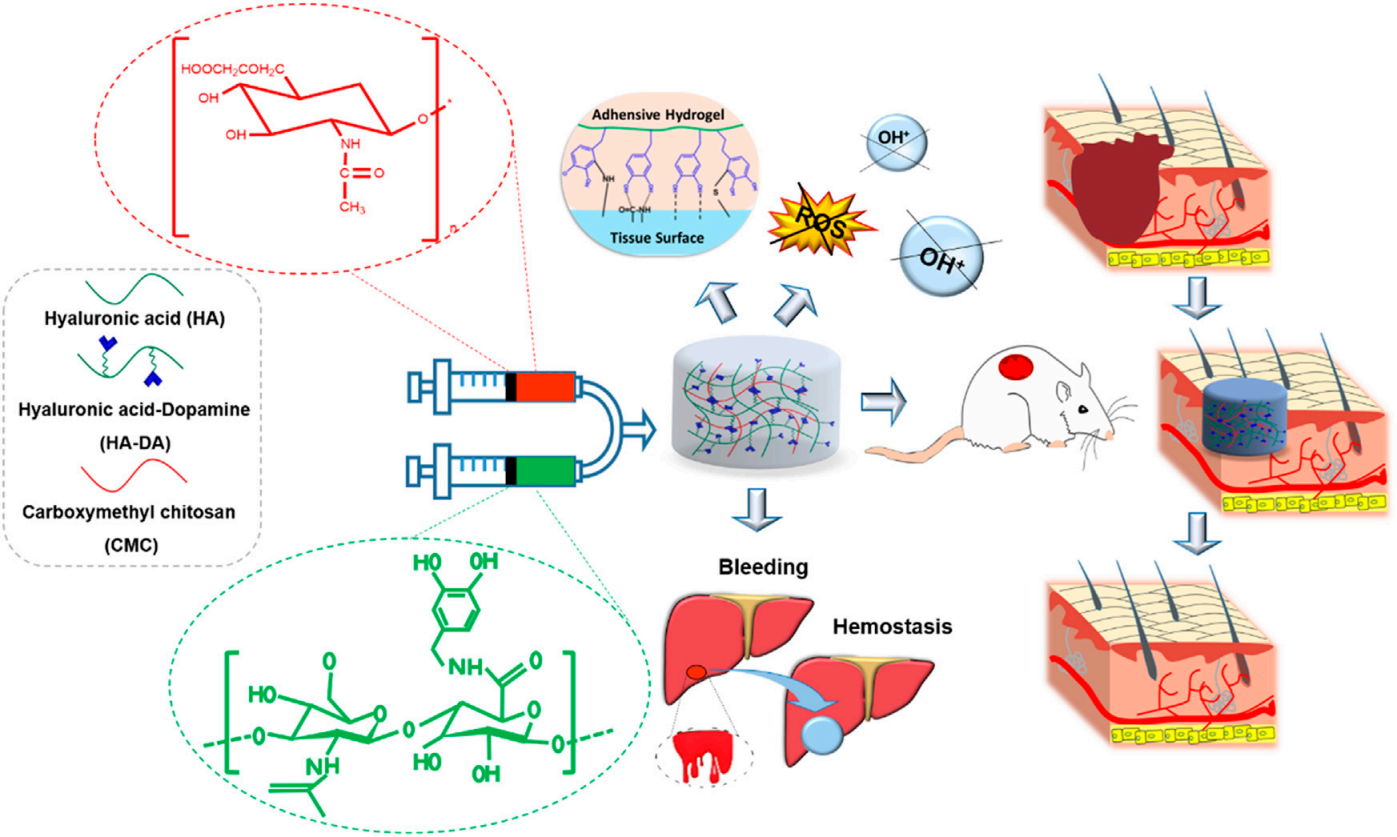
Schematic illustration of the CMC/HA-DA hydrogel for wound repairing: the CMC/HA-DA hydrogel was synthesized by the enzyme cross-linking method with HRP/H_2_O_2_ with its advantage of mild conditions; the CMC/HA-DA hydrogel not only has enhanced adhesive, antioxidant, and hemostatic ability but also was injectable, biodegradable, and biocompatible, which promise rapid wound closure (Cui et al., 2022).

Our previous study demonstrated an injectable multifunctional hydrogel prepared with HA, dopamine, and carboxymethyl chitosan (CMC/HA-DA) for repairing skin injury (Cui et al., 2022). This CMC/HA-DA hydrogel possessed excellent adhesive, antioxidant, and hemostatic properties which strongly contributed to promoting skin injury repair (Figure 3). Zhang et al. (2021) designed a balanced charged hydrogel with alginate, HA, and polylysine (PLL) to obtain anti-biofouling and antioxidant properties; after carrying the curcumin and epigallocatechin gallate (EGCG), the drug delivery hydrogel can significantly weaken the development of irradiation-induced skin injury. Leite and Frade (2021)compared saline, chlorhexidine digluconate, and 0.2% HA by covering the lesion of skin-abraded rats with sufficient amount, and the result indicated that 0.2% HA presented better healing efficacy of skin abrasions (Leite and Frade, 2021).

### 3.3 Hyaluronic Acid Used in Cancer Treatment

In some types of cancer, HA is used to monitor the progress because of its mucoadhesive property (Luo et al., 2019). HA, along with its derivatives, particularly combines with some cell surface receptors (Kim et al., 2019). These cell surface receptors commonly exist in regions such as kidneys, vessels, body fluids, liver, and mainly tumor tissues (Kim et al., 2018). These specific binding functions are important for drug delivery targeted HA, which promises loading and delivery of proteins, nucleic acids, peptides, and different anticancer agents. HA could also be conjugated with folic acid for tumor treatment delivery and anticancer transmission. After the discovery of HA, its benefits are highly appreciated for contribution to accurately recognizing overexpressed CD44 receptors. In the recognition of CD44 receptors, the stable particle hydrophallic with HA is used to enhance the killing of cancer cells. Tumor cells that expressed CD44 on their layers can also be used in the targeting of HA-based formulation (Kim et al., 2019). For example, for targeting cancer cells, HA was applied to decorate nano-liposomes, which showed a strong interaction with the cells of upregulated CD44 expression, in contrast with the cells of downregulated CD44 expression, and their nanoscales may promise potential applications in monitoring the insides of tumor cells (Voigt and Driver, 2012). Nowadays, practice in the treatment of cancer is an important element in medical fields. Different surgeries including radiation treatment and chemotherapy are used in cancer treatment. In addition, the therapies of gene delivery, targeted chemotherapy, and immunotherapy have also been tested in clinical and translational research. HA-based materials are highly used in cancer treatment to better cooperate with the aforementioned methods (Huang and Huang, 2018b). Significant outcomes in preclinical and *in vitro* studies had been reported by different groups relevant to drug-loaded nanoparticles with HA polymers to enhance the ability to target the tumor cells.

Presently, the potentials of HA in medication transfer have been explored as a carrier of antitumor and anti-inflammatory drugs. HA is reflected as one of the main components of the ECM; similarly, it is the foremost ligand for CD44 and RHAMM (Zhong et al., 2016), which are overexpressed in a range of tumor cell surfaces including colon cancer, human breast epithelial cells, lung cancer, and acute leukemia (Zamboni et al., 2017). Our previous work demonstrated that colorectal cancer and adjacent normal mucosa differed in apoptotic and inflammatory protein expression (Sun et al., 2022), and HA may play different roles in regulating colorectal cancer and adjacent normal mucosa. HA is crucial to the cure and termination of cancer cell metastasis and the localization of drugs not only to the tumorous cells but also to the nearby lymph nodes (Lee et al., 2020). HA is also well-known as a bioadhesive composite proficient in binding with great empathy to cell-surface and intracellular receptors, to the ECM components, and to the aforementioned cells. HA can impasse to receptors in tumor cells and is intricate in tumor evolution and scattering. CD44 regularizes cancer cell creation and metastatic evolutions. In anticancer drugs which have been used in the field of tumor therapy, HA has the capability to represent the receptor on the layer of tumor cells and can be used for better killing and to target anticancer drugs. In addition, the disruption of HA–CD44 binding was shown to reduce tumor progression. Also, the supervision of exogenous HA caused the halt in tumor dispersal. Consequently, anticancer medication stabilization, localization and solubilization, and controlled discharge could be improved by pairing with HA. Also, it is specified that a high HA level has been perceived at the offensive front of rising breast cancers, 3.3-fold greater than that in central positions inside the tumor. The overproduction of HA is linked with the reduced prediction of breast cancer. In females <50 years, breast cancer HA levels might forecast cancer reversion. It was described that HA conjugates comprised anticancer medicines containing sodium butyrate which showed enriched targeting capability toward cancer and advanced therapeutic efficiency linked to free-anticancer drugs dependent on the amount of exchange of HA with drugs. It is well known that cisplatin (cis-diaminedichloroplatinum or CDDP) is a widely engaged chemotherapeutic mediator for the cure of a widespread spectrum of solid cancer (Wiercińska et al., 2020). Cho et al. explained fruitful drainage of HA–cisplatin (HA–Pt) conjugates into the auxiliary lymph lumps with condensed systemic toxicities after being confined in osculation in a woman suffering from breast cancer. The results of their study found that the respiratory provision of the HA–Pt conjugate to the lungs might be beneficial in the cure of lung cancer by decreasing general toxicities and growing CDDP deposition and retaining inside lung tumors, nearby lung tissues, and in the meditational lymph (Cho, 2020).

### 3.4 Ophthalmology

HA is a normal element of the vitreous humor of the eye, and it has several effective uses in ophthalmologic surgical procedures. HA is predominantly beneficial as a space-filling matrix in the eye; therefore, intraocular injection of HA in operations is used to preserve the shape of the frontal chamber (Salzillo et al., 2016). HA solution can also be used as a tackifier of eye drops and an auxiliary agent for eye tissue repair (Huynh and Priefer, 2020). HA is used to relieve the eye through visual inspection, cataract extraction, ocular operation and anterior–posterior section operations, lens embedding, and vitreous retinal operation because of its protective nature toward the visible tissue, such as the corneal endothelium. Thus, HA is a significant facilitator in the reestablishment operation of eye parts. HA has been deliberated widely in its uses for the cure of diseases related to eye dryness which is related to the disease of ocular surface and tears, which results in indications of visual disorder, uneasiness, and tear film volatility. Between 5 and 34 percent of persons are affected by eye dryness, having signs ranging from burning, frontal body sensation, redness, photophobia, and stinging. At this time, the most general treatment is the usage of artificial tears prepared by using cellulose derivatives, hydroxypropyl guar, polyvinyl alcohol, and HA (Salwowska et al., 2016). These jointly have been revealed to raise tear film constancy, increase contrast sensitivity, and decrease surface pressure and visual surface excellence (Lu et al., 2018). HA exhibits viscoelastic possessions that are used to lubricate the optical surface by decreasing friction through blinking and ocular activities. Consequently, the water-retaining and lubricant possessions of HA are used openly to cure the diseases of eye dryness.

### 3.5 Hyaluronic Acid Application in Cosmetics

Skin problems such as nasolabial folds, wrinkles, skin hydration, collagen stimulator, anti-aging, and skin augmentation can be cured by using HA as a cosmeceutical. HA has been used in a wide range of cosmetic formulations because of its strong water-holding capability. HA can also be used to maintain the turgidity, moisture, and elasticity of skin (Dong et al., 2017). A study was carried out on 76 females aged 30–60 years with signs of periocular wrinkles. They were recommended with HA cream formulation to apply around the wrinkled area two times a day for 2 months. The HA-based cream showed incredible results in elasticity and skin hydration. For the targeted drug delivery system, HA has been used as a targeting ligand. We can increase the penetrability across biological membranes and also improve their efficiency of targeting by using HA-based modifications (Gomes et al., 2020). HA exhibits significant nutricosmetic and cosmetic efficacies in sorting out different skin defects such as skin aging, nasolabial folds, and wrinkles (Caló and Khutoryanskiy, 2015). To study nutricosmetic and cosmetic effects, HA has been used in different forms, that is*,* creams, serum, gels, lotion, intra-dermal filler injections, and facial fillers. The nutricosmetic and cosmetic effects of HA have been connected with its capability to introduce face rejuvenation, collagen stimulation, and tissue augmentation (Salwowska et al., 2016).

## 4 Biomaterials Prepared With Hyaluronic Acid

Because of the broad adaptation and biomedical needs, HA has been fabricated into many biomaterials for different application, such as coatings, injectable hydrogels, and nanoparticles.

### 4.1 Hyaluronic Acid Coatings

**FIGURE 4:**
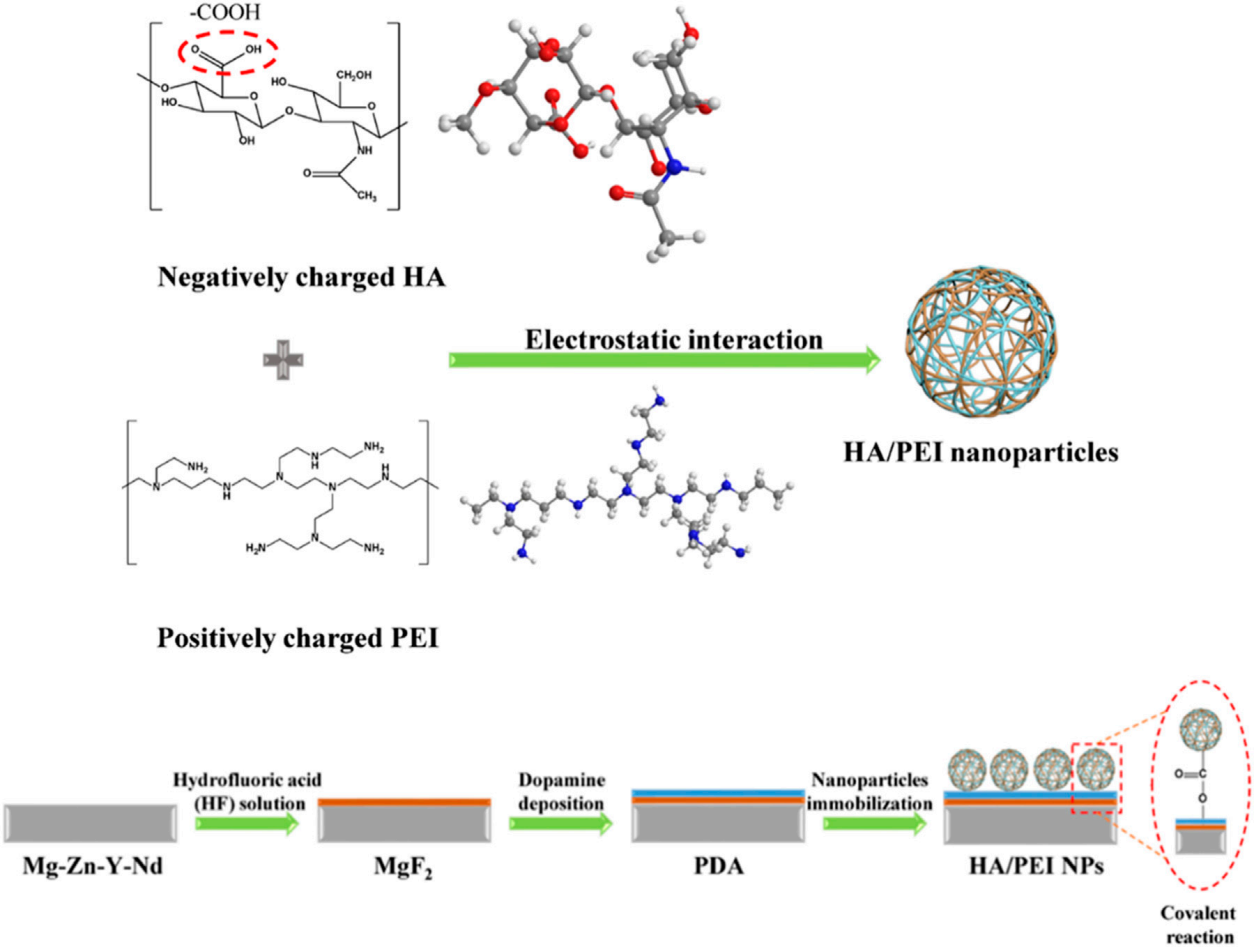
Surface modification of the Mg alloy with HA nanocomposite coating: nanoparticles prepared with HA and polyethyleneimine (PEI) by electrostatic interaction were conjugated onto the Mg-Zn-Y-Nd alloy which was pre-treated with hydrofluoric acid and dopamine solution (Li et al., 2021b).

HA is the skeleton component of the ECM and plays an important role in coordinating cell behavior, and therefore, in the early stage of application, it is often used as a natural material to improve the universal biocompatibility of implants, which is coated on the surface (Yu et al., 2021). The HA coating’s function usually depends on the density of the immobilized HA molecules (Wu et al., 2016). With the development of technology, many studies have reported that HA and other ECM components co-immobilized on the surface of materials will give them more and better functions (Tong et al., 2022). For instance, the coatings comprising HA and collagen will enhance blood compatibility, cell compatibility, and tissue compatibility of the materials (Li et al., 2016); the coatings comprising HA and heparin can not only improve the anticoagulant function of the implant surface but also relieve the hemolytic symptoms caused by the sudden release of heparin (Bai et al., 2020); and the coatings comprising HA and PD-1 can promote the endothelialization of injured vessels and inhibit the formation of hemangioma (Bai et al., 2021). Another important discovery in HA coating development is that the biological function of HA coating with different molecular weights may be reversed. There is no doubt that all HA coatings have the function of inhibiting platelet adhesion, but the coatings comprising HA with high molecular weight (> 10^5^ Da) inhibit macrophage adhesion/activation, whereas the coatings prepared with HA of low molecular weight (< 10^4^ Da) promote macrophage adhesion/activation. In the application of cardiovascular implants, 10^5^ Da was chosen as an optimized molecular weight to fabricate the HA coating, which will endow the surface with better blood compatibility, anti-hyperplasia, anti-inflammation, and pro-endothelialization ability (Li et al., 2017). Our previous work also found that HA coatings may absorb the degradation products of magnesium (Mg) alloy to coordinate the degradation behavior of the Mg alloy with the microenvironment to solve the problems of rapid degradation and delayed endothelialization of biodegradable Mg alloy stents (Li et al., 2020a). Another work found that HA nanoparticles were able to combine with the Mg ion and control the interaction of the Mg ion and EC, further improving EC growth and nitric oxide (NO) release (Wang et al., 2020a). Thus, the HA nanoparticles were conjugated onto the Mg alloy surface to design a nanocomposite coating (Figure 4), and this coating was proved to have a stronger ability for pro-endothelialization and anti-inflammation (Li et al., 2021b).

**FIGURE 5:**
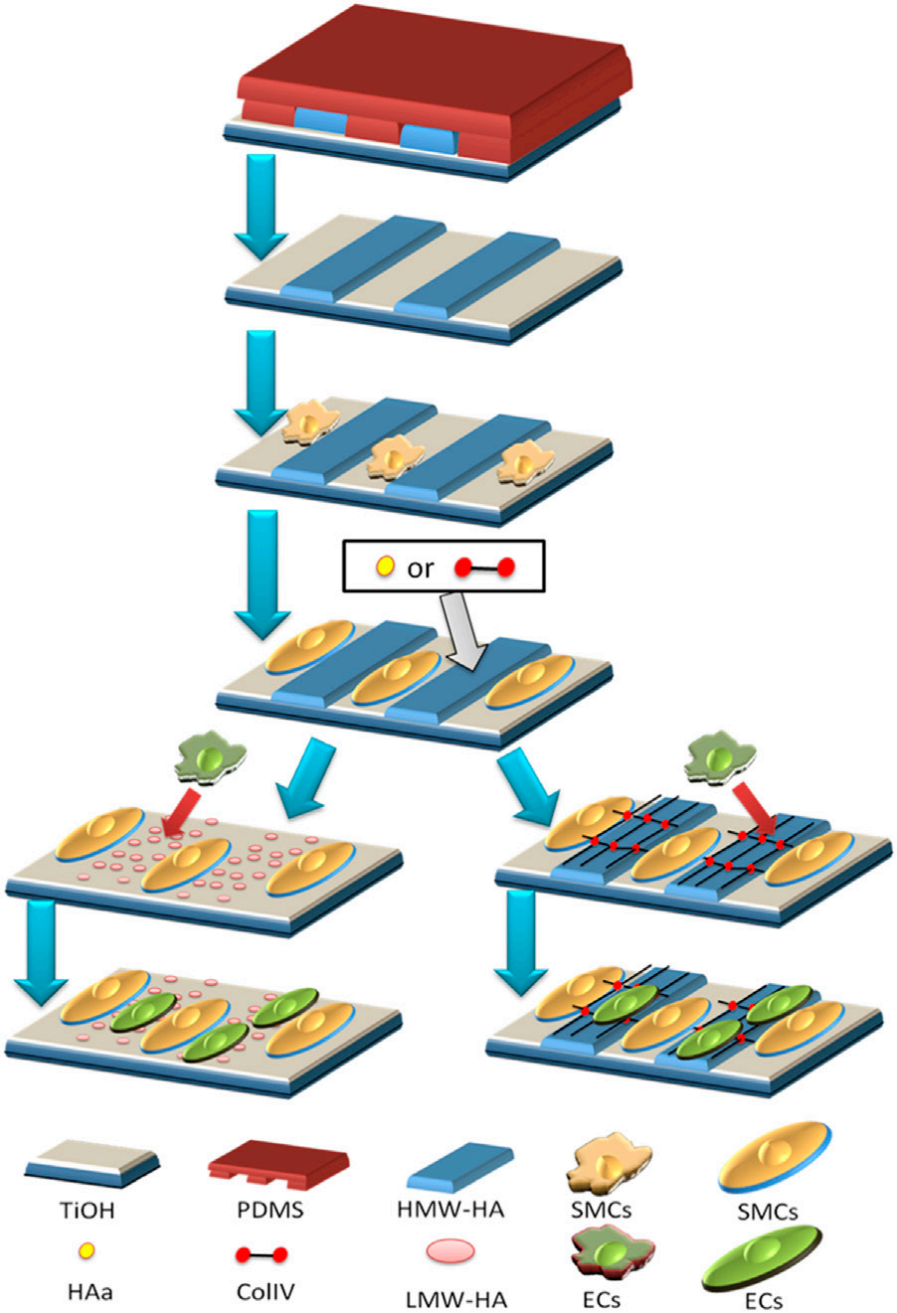
Two co-culture models of EC/SMC on the HA micro-pattern: SMC was first regulated to the contractile phenotype; in model 1, hyaluronidase was added to break down high molecular weight HA into low molecular weight HA, and the latter induced EC to grow on its surface; in model 2, type IV collagen was added to cover the HA micro-pattern and the patterned SMC, and then type IV collagen promoted EC to grow on its surface (Li et al., 2014).

Inspired by the inhibiting effect on cells of high molecular HA, HA of 10^6^ Da was prepared in micro-patterns of different sizes and was finally proved to possess a similar regulation effect of blood flow shear stress (15 dyn/cm^2^) on EC with pattern scales of 25 μm (Li et al., 2013). This HA micro-pattern also regulates the smooth muscle cells (SMCs) to a physiological state (contractile phenotype) (Li et al., 2014). Further co-culture models were also built on the HA micro-pattern, which verified that the contractile SMCs promote EC growth and functional factor release and finally promote the formation of the biomimetic vascular intima (Figure 5) (Li et al., 2014). These research studies also suggest that the HA micro-pattern covered with type IV collagen is a better cell template for EC/SMC co-culture, which provides inspiration for the HA/collagen coating design (Li et al., 2016).

**FIGURE 6:**
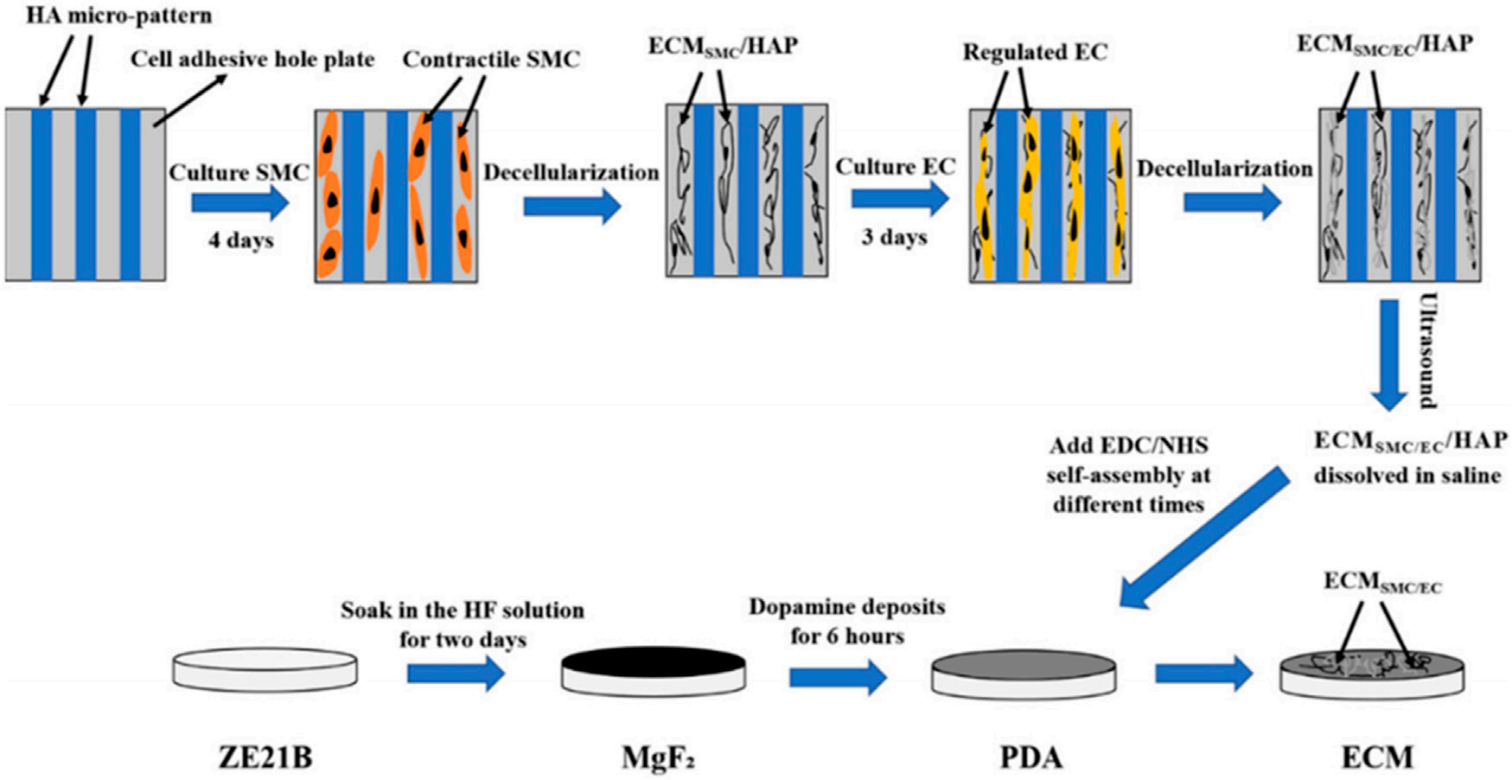
Fabrication of the biomimetic vascular basement membrane with the ECM secreted from the SMC and EC controlled by HA micro-patterned on the non-biodegradable and biodegradable material surfaces (Liu et al., 2022).

Inspired by another de-cellularized technology, the ECM secreted by the HA-patterned EC was deposited on the cardiovascular implanted material surface (Figure 6), which enhanced their biocompatibility (Zou et al., 2019). The structure and function of the vascular basement membrane provide more enlightenment, and the ECM secreted by the HA-patterned EC and SMCs endows the materials with stronger multi-functions than the single HA-patterned EC-ECM (Liu et al., 2022).

### 4.2 Hyaluronic Acid Nanoparticles

HA nanoparticles come from the clinical demand for nano-scaled HA, which depends on the specific interaction of HA and CD44 on the cell membrane. CD44 is often widely distributed in cancer cells and is regarded as a target for the carrier of loading anticancer drugs (Li et al., 2020b; Cui et al., 2020; Ding et al., 2021). The authors’ experience also displayed a story: Prof. Liu of Qiqihar University suffered from advanced pancreatic cancer, and his doctor diagnosed that he could not live for half a year. He contacted us for help because he could not stand the pain. Thus, he was suggested to try to use disulfiram and copper gluconate under the guidance of his doctor, which made him live for another year with high quality, and finally, he died of a cerebral hemorrhage after an accidental fall during a walk. This case encourages the authors to continue their efforts in the research and development of low-cost anticancer drug disulfiram. Subsequent studies focused on HA nanoparticles loaded with an appropriate proportion of disulfiram and copper ions, and the results showed that the drug loaded with this targeted carrier had a better therapeutic effect and lower negative cytotoxicity (Xu et al., 2021). In addition, HA nanoparticles have been proved to have good blood compatibility (Li et al., 2021b), which may also solve the complications of cerebral hemorrhages caused by disulfiram.

Another recent study demonstrated that the Mg ion could regulate the macrophages from inflammation phenotype (M1) to immune phenotype (M2), which contributed to EC growth and functionalization (Hou et al., 2022). Inspired by the study mentioned in Section 4.1, HA nanoparticles could combine with the Mg ion and carry more Mg ions to the cells, suggesting HA nanoparticles as a promising carrier for Mg drugs. However, the precondition is that the particle size after loading magnesium ions is less than 500 nm. On this basis, the smaller the particle size, the easier it will be for the nanoparticles to enter the cells. This technology may have potential applications in developing drugs or biomaterials for cardiovascular diseases or cerebrovascular diseases.

In addition, it has been reported that the HA nanoparticles have potential as nanomedicine in the treatment of inflammatory diseases (Rao et al., 2020). Kang et al. (2021)reported an empty self-assembled HA nanoparticle (HA-NP) as a potential therapeutic agent in osteoarthritis treatment: HA nanoparticles blocked the receptor-mediated cellular uptake of free HA with low molecular weight, and the cellular uptake of HA nanoparticles was increased by the ectopic expression of CD44, using an adenoviral delivery system; HA nanoparticles presented *in vitro* resistance to digestion with hyaluronidase and *in vivo* long-term retention ability in knee joint, compared with free HA with a high molecular weight. Lierova et al. (2020)found that HA nanoparticles could reduce harmful radiation-induced processes in lung tissue, thereby potentially decreasing ECM degradation leading to pulmonary fibrosis.

### 4.3 Hyaluronic Acid Hydrogels

HA hydrogels with excellent functions have wide biomedical applications, particularly in tissue engineering, wound healing, and drug delivery (Wang et al., 2020b; Guan et al., 2021; Wei et al., 2021; Wei et al., 2022). The function of HA hydrogels is usually influenced by their essential physiochemical properties, including molecular weight and functional group modification. However, there is no consensus on the best molecular weight for specific applications. It has been reported that using higher molecular weight HA leads to quicker gelation times and stiffer, more stable hydrogels with smaller mesh sizes, and this result indicates that the HA with a molecular weight of 5 × 10^5^ Dalton may be the optimal choice for a readily injectable, *in situ*-forming hydrogel with appropriate biophysical properties to promote vascular cell spreading and sustain vascular network formation *in vitro* (Browne et al., 2020). Mondal et al. (2016)also demonstrated that an injectable high molecular weight (1.2 × 10^6^ Dalton) HA hydrogel was a promising material for cartilage regeneration. Yet, our previous study showed that low molecular weight HA hydrogels had the advantage of promoting mesenchymal stem cell (MSC) growth, which made the hydrogels excellent carriers of MSC delivery, contributing to brain injury treatment (Zhang et al., 2018). Further adjusting the feeding ratios will control the gelation time, moisture content, modulus of elasticity, and degradation rate of the HA hydrogel to make it a better match with the tissue of the brain injury (Zhang et al., 2018). Then, by mixing high molecular weight HA and low molecular weight HA, will the hydrogels obtain functions of superposition? Xue et al. (2020)reported a hydrogel obtained from mixing both high and low molecular weight HA, which had stronger *in vitro* anti-degradation ability, better mechanical properties, and lower cytotoxicity, suggesting potential applications in regenerative medicine and tissue engineering. The function of the hydrogels was controlled by the ratio of the high molecular weight HA and low molecular weight HA (Xue et al., 2020).

Sulfonation is an effective chemical modification method of HA molecules, which can not only improve their stability *in vivo* but also greatly improve a variety of biological functions, such as anti-inflammatory, promoting physiological cell proliferation, and so on (Yu et al., 2020). Data showed that HA with a higher sulfonation degree had stronger functions of inhibiting inflammatory macrophages, promoting the adhesion, proliferation, and migration of EC and regulating and maintaining the contractile phenotype of SMCs (Yu et al., 2020), which is a promising potential application in vascular tissue engineering (Yu et al., 2020). Wu et al. (2021) used polyaniline and sulfonated hyaluronic acid to construct flexible hydrogels with simulated skin conductivity. *In vivo* results showed that the electrical stimulation method by this hydrogel was superior to the present clinical electrical stimulation strategy to promote the healing of infected chronic wounds.

In addition, the HA hydrogel itself or coupled with other substrates such as hydrophobic carbon nanodots, chitosan, alginate, and gelatin can be applied for wound dressing because of their excellent anti-inflammation, anti-bacterial property, and biocompatibility (Bazmandeh et al., 2020; Fotso Kamdem et al., 2021; Mauro et al., 2021; Meng et al., 2021).

## 5 Conclusion and Prospect

In this article, the relationship between the molecular properties of HA and the advances in biomedical applications was systematically explored. Biocompatibility based on the molecular weight makes HA widely used in all directions of tissue engineering. In particular, low molecular weight HA is favored for wound healing because of its positive effects on cell adhesion, proliferation, and apoptosis inhibition, but it may stimulate an inflammatory response. The targeted interaction with CD44 on the surface of tumor cells makes HA an ideal drug carrier in cancer treatment. Matching lubricity and elastic modulus and excellent biocompatibility ensures that HA becomes the perfect ophthalmic biomaterial. Super moisturizing and anti-aging properties determine that HA is an irreplaceable ingredient in the current cosmetics scenario. Multifunctional coatings, targeted nanoparticles, and injectable hydrogels as novel HA formulations strengthen its effect in the aforementioned applications. Sulfonation, as an available chemical modification technology, not only improves the stability of HA formulation but also greatly improves its biological properties.

The future application of HA may focus on composite biomedical platform systems based on sulfonated HA nano-hydrogels, which integrate the time and space of the orderly release of multifunctions including targeted tracking, controlled drug delivery, and precise regulation of different cells as an organic whole. These composite biomedical platform systems will give full play to the respective advantages of new formulations of HA, which require strict standards for the corresponding parameters. The present frontier research shows some preliminary data: HA with molecular weight ranging from 10^5^–10^6^ Dalton can both inhibit inflammation and promote tissue regeneration; a higher sulfonation degree of HA leads to better stability and biocompatibility, which may form smaller nanoparticles; HA nanoparticles with smaller sizes have higher delivery efficiency because they can cross the cell membrane faster. These findings gradually make it possible to develop the expected composite biomedical platform systems of HA.
